# Efficacy and Safety of Elobixibat in Parkinson's Disease with Chronic Constipation: CONST‐PD Study

**DOI:** 10.1002/mdc3.13972

**Published:** 2024-01-24

**Authors:** Taku Hatano, Genko Oyama, Yasushi Shimo, Kotaro Ogaki, Noriko Nishikawa, Ryota Nakamura, Taiji Tsunemi, Takashi Ogawa, Hiroto Eguchi, Kensuke Daida, Naohide Kurita, Shin‐ichi Ueno, Jiro Fukae, Wataru Sako, Kenta Shiina, Sho Nakajima, Yutaka Oji, Ryo Wakamori, Shinji Saiki, Kenya Nishioka, Ayami Okuzumi, Daisuke Taniguchi, Haruka Takeshige‐Amano, Atsuhito Fuse, Asuka Nakajima, Masayoshi Kano, Hikaru Kamo, Yuri Yamashita, Atsuhiko Shindo, Naotake Yanagisawa, Nobutaka Hattori

**Affiliations:** ^1^ Department of Neurology Juntendo University Faculty of Medicine Tokyo Japan; ^2^ Department of Neurology Juntendo Nerima Hospital Tokyo Japan; ^3^ Department of Neurology Juntendo Urayasu Hospital Urayasu Japan; ^4^ Juntendo Clinical Research and Trial Center Juntendo University Hospital Tokyo Japan

**Keywords:** movement disorders, Parkinson's disease, autonomic, randomized trials, clinical neurology, constipation

## Abstract

**Background:**

Chronic constipation is a common digestive complication of Parkinson's disease (PD).

**Objectives:**

To verify the usefulness of elobixibat, an ileal bile acid transporter inhibitor, for chronic constipation in PD.

**Methods:**

This double‐blind, placebo‐controlled study consisted of a 2‐week observation/washout period and a 4‐week treatment period. All patients received a Bowel Movement Diary at Week ‐2 and were allocated to elobixibat (10 mg) or placebo at Week 0. Patients visited at Weeks 2 and 4 to report daily spontaneous bowel movements (SBM), stool form, drug use, quality of life (QOL), and safety. Changes in these parameters were assessed.

**Results:**

The study included 38 patients in the elobixibat group and 39 in the placebo group, and 37 each completed the study. SBM frequency/week (mean ± standard deviation) increased significantly from 4.2 ± 2.6 at baseline to 5.9 ± 3.2 at Week 4 in the elobixibat group (*P* = 0.0079), but not in the placebo group (4.5 ± 2.7 to 5.3 ± 3.5; *P* = 0.0889). On analysis of covariance, the between‐group difference in frequency changes at Week 4 (primary endpoint) was not significant after adjustment by baseline and sex (point estimate = 0.8; 95% confidence interval = −0.57 to 2.09, *P* = 0.2601), although a significant difference (*P* = 0.0011) was evidenced at Week 1 by a similar analysis. Stool form and scores of satisfaction and stigma were improved by elobixibat. Adverse events were as previously reported.

**Conclusions:**

Elobixibat improved the SBM frequency, though the defined primary endpoint was not evidenced. QOL parameters (stool consistency and treatment satisfaction) were also improved. Elobixibat may have therapeutic benefits in PD patients suffering from chronic constipation.

**Trial Registration Information:**

Trial Registration Number: JPRN‐jRCTs031200172 (submitted: October 26, 2020; first patient enrolment: December 23, 2020; https://jrct.niph.go.jp/en-latest-detail/jRCTs031200172).

Parkinson's disease (PD) is one of the most common movement disorders caused by dopaminergic neuronal degeneration. Various non‐motor symptoms, including dementia, anxiety, sleep disturbance, and autonomic dysfunction occur occasionally in PD patients and decrease their quality of life (QOL).^1^ Chronic constipation is a common digestive complication in the prodromal/early phase as well as at a later stage of disease progression.^2^, ^3^, ^4^, ^5^, ^6^, ^7^, ^8^ Constipation may be life‐threatening if long‐lasting, causing intestinal perforation and/or megacolon syndrome, and should therefore be appropriately controlled,^9^, ^10^ using, for example, macrogol and lubiprostone, which are considered “likely efficacious” and rated as “possibly useful” for cases in which dietary approaches have been ineffective.^11^

Elobixibat (Elo), an ileal bile acid transporter (IBAT) inhibitor, has recently become available for chronic constipation. IBAT, expressed in the distal ileum, mediates reabsorption of bile acids (BAs) into the liver. The levels of BAs are increased in intestinal lumen if IBAT activity is suppressed, causing water and electrolyte influx into the lumen.

BAs also interact with transmembrane G protein‐coupled receptors, triggering serotonin release, activating the intrinsic, afferent neurons and motor neurons, enhancing large‐intestinal motility^12^ and inducing colonic high‐amplitude propagated contraction.^13^ Thus, IBAT inhibition is an appropriate strategy for the treatment of chronic constipation.^14^ Given this concept, a randomized, double‐blind, phase 3 study and its subsequent long‐term study showed that Elo resolved idiopathic chronic constipation with no serious safety concerns.^15^ Therefore, we hypothesized that Elo is useful for treating PD‐related chronic constipation and conducted a multicenter, randomized, placebo‐controlled, double‐blind, parallel‐group study (JPRN‐jRCTs031200172) of Elo to evaluate its efficacy and safety in PD patients with chronic constipation.^16^

## Methods

### Overall Study Design

The study protocol and statistical analysis plan were previously published.^16^ Briefly, it consisted of a 2‐week observation/washout period and a 4‐week treatment period. At the beginning of the observation period (Week −2/Visit 1), patients were temporarily registered based on the inclusion/exclusion criteria^16^ referring to the Rome IV criteria^17^, ^18^ after providing written, informed consent. Patients with malignant tumors or organic constipation were excluded. Those who had received gastrointestinal surgeries or had gastrointestinal disorders were also excluded by the physician's judgment. Bowel Movement Diary (BMD) was provided to each patient, and the patients were instructed to daily record drug use, bowel movements (BMs), etc. in the BMD. At Week 0/Visit 2 (end of the observation period), the patients were assessed based on the patient criteria for final study inclusion. Drug allocation to either Elo or its indistinguishable, matched placebo (Pbo) was based on a stratified, permuted block method with sex as an allocation factor. Eligible patients were randomized and double‐blinded via an Interactive Web Response System (IWRS).^16^

They started receiving two tablets of either Elo (=10 mg) or Pbo at Week 0. Once‐daily, preprandial intake of the investigational drug was scheduled during the 4‐week treatment period. The patients visited their institutional sites twice more (Week 2/Visit 3 and Week 4/Visit 4).^16^ One‐tablet dose adjustment was allowed at the discretion of investigator or subinvestigator, hereinafter collectively termed (sub)investigator, or individual patient, in case of no spontaneous bowel movements (SBMs), or excessive movements or discomfort observed during the next 24 h. Patients were allowed to interrupt intake of the investigational drug due to an adverse event (AE).

Concomitant medications were given to the patients for parkinsonism throughout the study period, except for Duodopa pump therapy (Abbvie, North Chicago, IL, USA) with levodopa/carbidopa hydrate intestinal gel. Bisacodyl suppositories (10 mg, once daily; given at Week −2) were allowed for rescue purpose in case of no bowel movement for ≥72 consecutive hours, and no other agents for constipation were allowed in this study.^16^ Prohibited drugs or measures were defined throughout the entire period to avoid potential effects on the study results and data interpretation.^16^

### Measurements

Each patient recorded BMs in the BMD daily. The primary study endpoint was weekly SBM frequency with no need for rescue therapy. Changes in mean SBM frequency from baseline (Week 0; from −6 to 0 days) to the final week (Week 4) were compared between the Elo and Pbo groups. The secondary endpoints included weekly changes up to Week 4 in the frequency of SBMs and complete SBMs (ie, no sensation of incomplete evacuation), stool form based on the Bristol scale (BSFS),^19^ and use of rescue medication. Patients’ QOL was surveyed at Weeks 0 and 4 using multiple forms of questionnaires, including the Japanese version of the Patient Assessment of Constipation Quality of Life (JPAC‐QOL),^20^ Movement Disorder Society‐unified Parkinson's Disease Rating Scale (MDS‐UPDRS),^21^ Parkinson's Disease Questionnaire‐39 (PDQ‐39),^22^ and Euro‐Qol 5 dimension‐5 level (EQ‐5D‐5L).^23^, ^24^ MDS‐UPDRS was assessed in an on‐medication state. Use of dopamine agents was also monitored, since improvement in constipation was expected to improve intestinal absorption of anti‐Parkinsonian agents.

These parameters were further subjected to subgroup analyses by the presence or absence of any other complications, age (≥ or <65 years), Hoehn and Yahr stage (1–4), duration of the underlying disease (≥ or <median = 6 years), L‐dopa equivalent daily dose (LEDD; ≥ or <median = 560 mg) used prior to (from −6 to 0 days) Elo or Pbo initiation, and duration of chronic constipation (≥ or <20 years).

### Safety Information

Vital signs and laboratory measures were determined at Weeks 0 and 4. Subjective symptoms and objective findings were collected at Weeks 0, 2, and 4. AE information was collected throughout the study period (Week −2 through Week 4). Discontinuation and interruption of the investigational drugs were also followed.

### Statistical Analyses

Based on Elo versus Pbo assessments performed in the previous clinical trials,^15^, ^25^, ^26^ a sample size of 40 patients for each group was estimated, with 90% detection power at a two‐sided significance level of 5%.^16^ Whereas the full analysis set (FAS), per‐protocol set, and safety analysis set were previously defined,^16^ the same population was included in each set; therefore, the FAS data are presented. Summary statistics including safety information were tabulated, and the primary and secondary endpoints were compared between the Elo and Pbo groups. Stool form was collected from the BMD, with weekly assessment for each patient as the median of 7‐day recorded BSFS types (1 to 7). Contrary to the worldwide trend,^27^, ^28^, ^29^ the morbidity of PD is higher in women than in men in Japan.^30^, ^31^, ^32^, ^33^, ^34^ Therefore, comparisons between the two groups for the primary outcome were made by analysis of covariance (ANCOVA) models using baseline values and sex as covariates and mixed effect models for repeated measures (MMRM) as a sensitivity analysis. Missing values were imputed by the last observation carried forward (LOCF) method only for Week 4 SBM and complete SBM assessments, but not for secondary assessments; therefore, patient numbers varied slightly depending on the assessment procedure, as seen in the Results section. Within‐group variations were assessed by paired *t*‐tests.

### Standard Protocol Approval, Patient Consent, and Study Registration

Ethics were addressed as described previously.^16^ This study was approved by the Juntendo University Certified Review Board (CRB3180012) and conducted in accordance with the Declaration of Helsinki, the Clinical Trials Act of the Japan Ministry of Health, Labour and Welfare, and related laws and regulations.

The participating patients were informed of the study details and provided their written, informed consent. This study was registered in the Japan Registry of Clinical Trials (JPRN‐jRCTs031200172).

## Results

**Figure 1:**
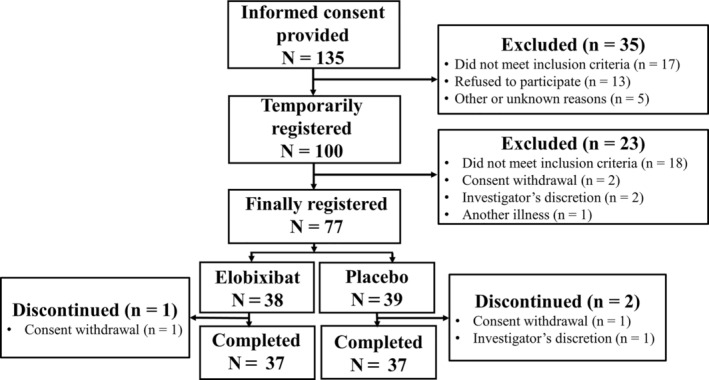
Flow chart of patient selection. Of the 135 patients who provided informed consent, 35 were excluded because they did not meet the inclusion criteria, etc., and the remaining 100 were temporarily registered. Twenty‐three patients were further excluded, leaving 77 for final registration at baseline (Week 0). Seventy‐four patients (37 each) completed the study. Elo, Elobixibat group; Pbo, Placebo group.

This study was conducted from October 22, 2020 through November 30, 2022. As shown in Figure 1, 100 of 135 patients who provided consent were temporarily registered; 23 were then excluded due to study ineligibility (18), consent withdrawal (2), investigators’ discretion (2), and a psychiatric problem (1), leaving 77 at final registration (38 and 39 in the Elo and Pbo groups, respectively). Of them, 74 (37 each) completed the study. The reasons for 3 discontinuations were consent withdrawal (2; 1 in each group) and investigator's discretion (1 in the Pbo group). Table 1 summarizes the enrolled patients’ demographic characteristics. There were no differences between the groups, including severity (Hoehn and Yahr stage), duration and treatment history of PD and associated chronic constipation.

**TABLE 1:** Patients’ demographic characteristics

Group	Elobixibat	Placebo
Number of patients	38	39
Sex, *n* (%)		
Male	18 (47.4)	19 (48.7)
Female	20 (52.6)	20 (51.3)
Age at consent (y)		
Mean (SD)	68.6 (8.6)	63.7 (9.2)
Height (cm)		
Mean (SD)	158.80 (10.63)	161.42 (9.18)
Weight (kg)		
Mean (SD)	58.94 (11.48)	59.59 (11.35)
Hoehn and Yahr stage, n (%)		
1	3 (7.9)	1 (2.6)
2	25 (65.8)	35 (89.7)
3	7 (18.4)	3 (7.7)
4	3 (7.9)	0 (0.0)
Duration of PD (y)		
Mean (SD)	7.8 (6.1)	7.8 (4.7)
Duration of PD categorized (y), n (%)		
<1	0 (0.0)	0 (0.0)
≥1–<5	15 (39.5)	10 (25.6)
≥5–<10	10 (26.3)	13 (33.3)
≥10–<20	10 (26.3)	15 (38.5)
≥20	3 (7.9)	1 (2.6)
Duration of chronic constipation categorized (y), n (%)		
<1	0 (0.0)	3 (7.7)
≥1–<5	8 (21.1)	10 (25.6)
≥5–<10	11 (28.9)	9 (23.1)
≥10–<20	12 (31.6)	10 (25.6)
≥20	7 (18.4)	6 (15.4)
PD treatment history, n (%)		
LEDD	38 (100.0)	39 (100.0)
Anti‐cholinergic	3 (7.9)	3 (7.7)
Droxidopa	0 (0.0)	0 (0.0)
Zonisamide	11 (28.9)	12 (30.8)
Istradefylline	3 (7.9)	6 (15.4)
Surgical treatment	1 (2.6)	3 (7.7)
Chronic constipation treatment history, *n* (%)		
None	7 (18.4)	11 (28.2)
OTC products	4 (10.5)	2 (5.1)
Osmotic agents	19 (50.0)	14 (35.9)
Stimulants	13 (34.2)	17 (43.6)
Epithelial function transformation drugs	4 (10.5)	3 (7.7)
Oriental medicines	2 (5.3)	2 (5.1)
Other medical history		
+	12 (31.6)	15 (38.5)
−	26 (68.4)	24 (61.5)
Complications*		
+	31 (81.6)	28 (71.8)
−	7 (18.4)	11 (28.2)

*Note*: Per‐week LEDD (mean ± SD) was 665.1 ± 508.1 mg/day and 733.8 ± 435.3 mg/day, respectively, in the Elo and Pbo groups.

Abbreviations: LEDD, levodopa‐equivalent daily dose; OTC, over‐the‐counter; PD, Parkinson's disease; SD, standard deviation.

* Data on the following categories of complications were collected: diabetes, kidney disease, liver disease, biliary tract disease, history of laparotomy, and others. Specific complications in each group were as follows.

Elobixibat group: hypertension, 11; hyperlipidaemia, 10; diabetes mellitus, 7; other complications, 57. Other complications that numbered ≥3 cases: insomnia, 4; spinal stenosis, 3; lumbar spinal stenosis, 3.

Placebo group: hypertension, 5; hyperlipidaemia, 8; diabetes mellitus; 6: other complications, 51. Other complications that numbered ≥3 cases: reflux oesophagitis, 4; insomnia, 3; lumbar spinal stenosis, 3.

### Effectiveness Endpoints

**Figure 2:**
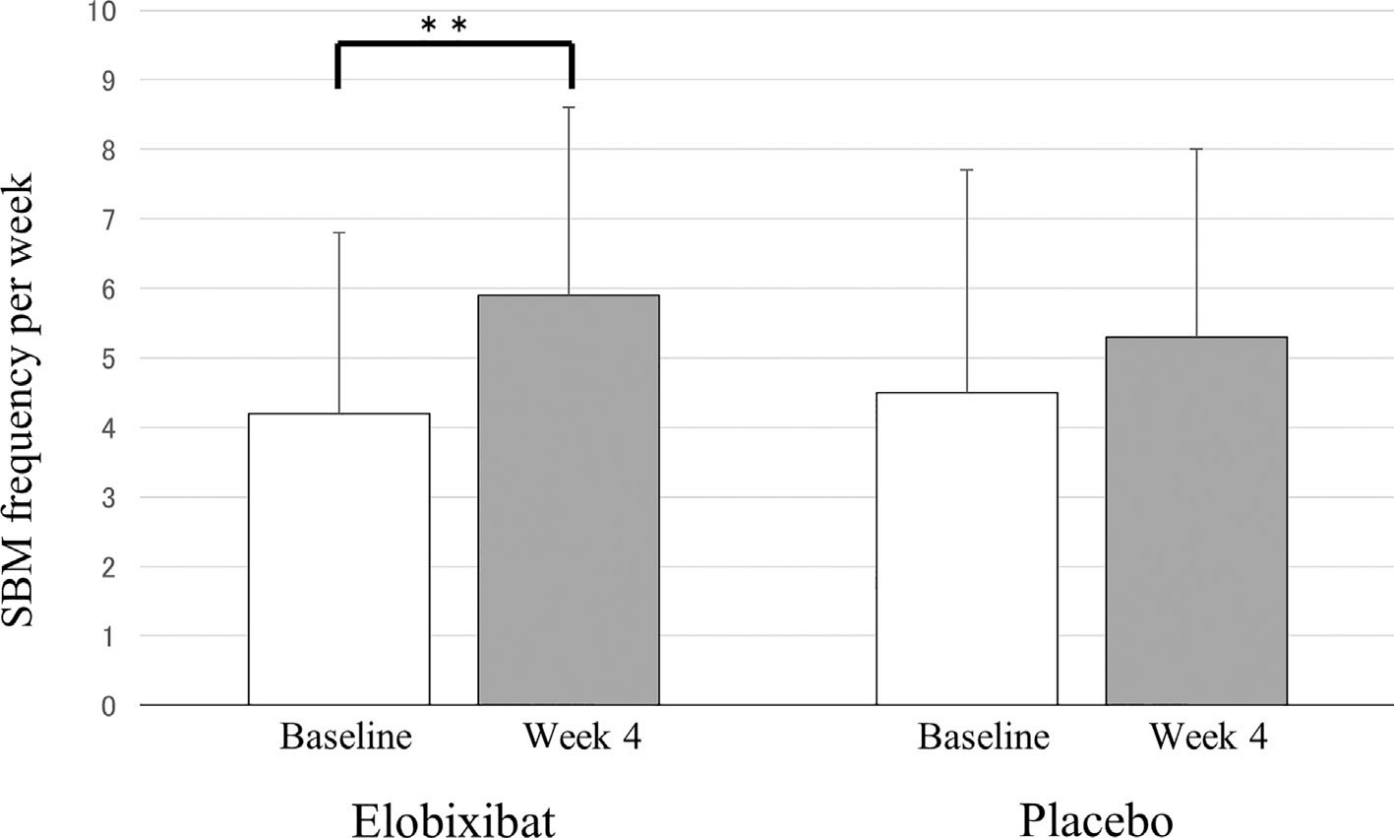
Primary endpoint: from‐baseline changes at Week 4 in the frequency of spontaneous bowel movement. Based on the BMD records of each patient, mean SBM frequency per week was calculated at baseline (Week 0) and Week 4 in the Elo and Pbo groups. ANCOVA analysis was performed for the comparison of SBM changes between the two groups, and paired *t*‐test was used to assess Week 4 versus baseline changes within each group. A significant difference is observed only for the Week 4 versus baseline change in the Elo group (**P = 0.0079). ANCOVA, analysis of covariance; BMD, bowel movement diary; Elo, elobixibat; Pbo, placebo; SBM, spontaneous bowel movement.

The weekly SBM frequency (mean ± standard deviation (SD)) changed from 4.2 ± 2.6 at baseline to 5.9 ± 3.2 at Week 4 in the Elo group, indicating a significant increase of 1.7 ± 3.7 (*P* = 0.0079) (Fig. 2). A slight, but insignificant, increase (0.8 ± 2.8; *P* = 0.0889) from 4.5 ± 2.7 to 5.3 ± 3.5 was seen in the Pbo group. After adjustment by baseline frequency and sex, ANCOVA analysis estimated the between‐group difference in SBM changes at Week 4 to be 0.8 (95% confidence interval (CI) = −0.57 to 2.09; *P* = 0.2601). With sex excluded as an adjustment factor, the between‐group difference in SBM changes at Week 4 was 0.77 (95% CI = −0.52 to 2.06; *P* = 0.2596), an almost equivalent result. The MMRM method similarly showed an insignificant between‐group difference (point estimate = 0.1, 95% CI = −0.12 to 0.31; *P* = 0.3742).

The results of the subgroup analysis performed for the primary endpoint by the presence or absence of any other complications, age, Hoehn and Yahr stage, PD duration, prior LEDD, and duration of chronic constipation are shown in Table S1. The subgroup analysis showed a significant difference in SBM frequency between treatment groups for patients aged <65 years, Hoehn and Yahr stage I (only 4 patients), and receiving prior LEDD < the median (Table S2).

An additional subgroup analysis was performed by baseline stool form. The weekly SBM frequency (mean ± SD) increased significantly from 4.1 ± 1.9 to 6.4 ± 3.3 at Week 4 in the Elo group patients with baseline BSFS type 1/2 (*P* = 0.0099; *n* = 18), whereas no significant changes in SBM frequency were observed in the Elo group patients with type 3–5 baseline stool form from 4.5 ± 3.2 to 5.7 ± 3.1 (*P* = 0.2441; *n* = 19). No significant changes were observed in these parameters in the Pbo group, from 4.4 ± 2.7 to 5.4 ± 4.1 (*P* = 0.2214; *n* = 17) and from 4.6 ± 2.7 to 5.2 ± 3.0 (*P* = 0.2567; *n* = 22), respectively.

Summary statistics of the secondary endpoint of weekly changes in SBM and complete SBM frequency from baseline are shown in Table S3, Figure S1(A,B).

### Stool Form

A total of 18 and 17 patients reported BSFS type 1 or 2 stool at baseline in the Elo and Pbo groups, respectively. At Week 4, the type 1/2 stool forms changed to type 3–5 (normal stool) in 10 (59%) Elo group patients and to type 6 (soft stool) in 2 (12%), whereas 5 (29%) still reported type 1 or 2 stools. On the other hand, type 1/2 stool forms changed to type 3–5 in 6 (35%) and did not apparently change in 11 (65%) at Week 4 in Pbo group patients.

**Figure 3:**
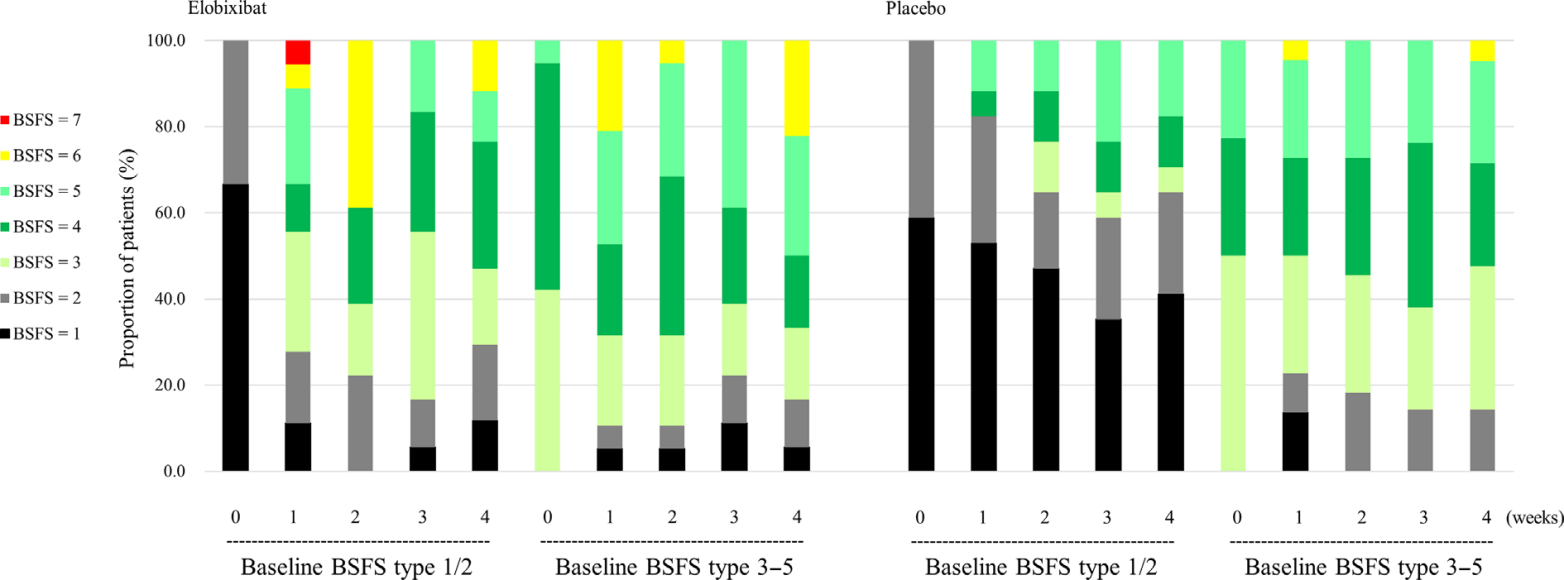
Weekly assessment of stool form. Stool forms were assessed for each patient as the weekly median BSFS (type 1–7). Patients subgrouped according to their baseline stool forms of type 1/2 and 3–5 were followed up in later weeks. The percentage of patients is shown with different colors corresponding to the weekly assessed median BSFS. 0 week is the baseline. Left panel: Elobixibat group; Right panel: Placebo group. BSFS, Bristol stool form scale.

Since about half of study patients reported type 3–5 stool forms at baseline, the changes in stool forms were further analyzed by baseline types 1/2 and 3–5. In the Elo group, change from type 1/2 to type 3–5 was observed in 11 patients and even from type 3–5 to type 6/7 in 4 patients at Week 1 (Fig. 3). A similar pattern continued up to Week 4. In the Pbo group, only 3 of 17 type 1/2 patients changed their stool forms to type 3–5 at Week 1, and 6 reported type 3–5 stools at Week 4. Handling the BSFS scale results as continuous values, the Wilcoxon rank‐sum test showed that the between‐group difference was significant at Weeks 1 and 2 (Table S4).

### Effects on Quality of Life

Multiple QOL indices (JPAC‐QOL, MDS‐UPDRS, PDQ‐39, EQ‐5D) were used to evaluate the 37 patients in each group, and the JPAC‐QOL satisfaction and PDQ‐39 stigma subscales were significantly better in the Elo group at Week 4 (Table 2). On the other hand, a significant improvement was observed for Pbo over Elo in MDS‐UPDRS Part II and total scores (Table 2). No other indices showed significant differences between the groups (also see Table S5).

**TABLE 2:** Changes in QOL scales from baseline to week 4

QOL scale	Subscale	Elo	Pbo	*P* value*
JPAC‐QOL	Physical Discomfort	−0.3 ± 0.8	0.1 ± 0.7	0.0518
	Psychosocial Discomfort	−0.1 ± 0.5	−0.1 ± 0.4	0.8281
	Worries/Concerns	−0.1 ± 0.6	−0.1 ± 0.5	0.8837
	Satisfaction	−0.7 ± 1.2	0.0 ± 0.8	0.0054
	Total	−0.2 ± 0.6	0.0 ± 0.4	0.1534
MDS‐UPDRS	Part I subtotal	−0.9 ± 2.3	−0.5 ± 2.7	0.4378
	Part II subtotal	1.1 ± 2.8	−0.4 ± 3.3	0.0426
	Part III subtotal	−1.1 ± 6.7	−4.0 ± 6.8	0.0701
	Part IV subtotal	0.1 ± 1.1	0.1 ± 0.7	0.9027
	Total	−0.9 ± 8.0	−4.8 ± 8.7	0.0485
PDQ‐39	Mobility	−0.7 ± 6.4	−1.5 ± 5.0	0.5577
	Activity of Daily Living	−0.1 ± 2.3	−0.6 ± 3.6	0.5389
	Emotional Well‐being	−0.6 ± 2.9	0.0 ± 3.6	0.4420
	Stigma	−0.6 ± 1.9	0.4 ± 1.8	0.0248
	Communication	−0.2 ± 1.1	−0.2 ± 0.9	1.0000
	Bodily Discomfort	−0.1 ± 1.7	−0.1 ± 1.4	0.9419
	Social Support	−0.1 ± 1.8	−0.1 ± 1.4	0.8857
	Cognition	0.0 ± 2.5	0.3 ± 2.1	0.6166
	Total	−2.4 ± 13.0	−1.8 ± 12.8	0.8434
EQ‐5D	Index Value	−0.0 ± 0.1	0.0 ± 0.1	0.0598
	VAS	−3.8 ± 15.3	0.7 ± 14.6	0.1961

*Note*: Values in the treatment group columns are expressed as means ± SD.

Abbreviations: Elo, elobixibat; EQ‐5D, Euro‐Qol 5 dimensions; JPAC‐QOL, Japanese version of Patient Assessment of Constipation Quality of Life; MDS‐UPDRS, Movement Disorder Society‐unified Parkinson's Disease Rating Scale; Pbo, placebo; PDQ‐39, Parkinson's Disease Questionnaire‐39; QOL, quality of life; SD, standard deviation; VAS, visual analogue scale.

* *P* values were based on the 2‐sample *t‐*test. The number of patients evaluated was 37 in each group.

A subgroup analysis of the JPAC‐QOL survey by baseline stool form showed a significant improvement of the satisfaction score in Elo group patients with BSFS type 3–5 (Table S6). In patients with baseline BSFS type 1/2, the baseline scores of worries/concerns, satisfaction, and total were worse in the Pbo group than in the Elo group, but no such tendency was observed in patients with baseline BSFS type 3–5 (Table S7).

### Dose Changes of Study Drugs

Among the 38 Elo group patients and 39 Pbo group patients in the FAS, the dose was not changed in 10 (26.3%) and 8 (20.5%), respectively, throughout the study period. A dose increase was observed in 12 (31.6%) Elo group patients, but the percentage (59.0%; 23 patients) was higher in the Pbo group, as expected. On the other hand, a dose decrease was observed in 9 (23.7%) and 2 (5.1%) patients, respectively. In 7 (18.4%) and 6 (15.4%) patients, study drugs were both increased and decreased.

### Use of Rescue Medicine

According to the rescue medicine defined in the previous report,^16^ none of the 77 enrolled patients concomitantly used any agents for constipation other than bisacodyl suppositories (10 mg). Of the 38 Elo group and 39 Pbo group patients, 11 (28.9%) and 8 (20.5%), respectively, used rescue medicine prior to study start, whereas 11 (28.9%) and 11 (28.2%) patients, respectively, used the medicine during the treatment period, with no between‐group difference. Mean frequency of the rescue medicine use was 1.3 times/week in both groups at baseline, while 1.6 and 1.5 times/week at Week 2 and 1.0 and 1.5 times/week at Week 4, respectively.

### L‐Dopa Equivalent Daily Dose for the Treatment of Underlying Disease

LEDD was summarized during the study period. The dose (mean ± SD in mg/week) was 665.1 ± 508.1, 664.0 ± 507.7, 661.1 ± 507.1, 678.1 ± 505.4, and 675.7 ± 511.8 in the Elo group, and 733.8 ± 435.3, 733.8 ± 435.3, 733.8 ± 435.3, 733.8 ± 435.3, and 737.5 ± 441.7 in the Pbo group at baseline, Weeks 1, 2, 3, and 4, respectively. The dose did not change markedly from baseline to Week 4 in both groups, though it appeared slightly lower in the Elo group.

### Safety Profile

AEs were observed in 21 Elo and 4 Pbo group patients. Table S8 summarizes the safety profile, with all reported AEs categorized into the System Organ Class of Gastrointestinal disorders. There were no AEs reported prior to study start. In the Elo group patients, soft feces and diarrhea were the most frequent AEs, and reported AEs including abdominal pain and malabsorption were likely associated with the underlying mechanism of the drug action. One Elo group patient who developed malabsorption reported repeated abdominal pain, diarrhea, and constipation. No adverse events or adverse drug reactions were observed, leading to discontinuation of the investigational drugs or requiring any treatments and/or hospitalization.

## Discussion

Chronic constipation is one of the major complications occurring in PD patients. Of the multiple classes of laxatives available, macrogol and lubiprostone have exceptional evidence showing their effectiveness for PD‐associated constipation.^35^, ^36^ Given the wide use of Elo for diabetes‐associated chronic constipation,^37^ we considered using it to treat PD‐associated constipation, and conducted the present randomized, Pbo‐controlled study. While anti‐cholinergic and dopaminergic agents that may influence the bowel movements were allowed in this study with dose modification, only three patients in each of the two groups used anti‐cholinergic agents; therefore, their data were not likely to largely perturb the overall assessments. The results showed a good effect of Elo, since per‐week SBM frequency increased significantly from 4.2 ± 2.6 at baseline to 5.9 ± 3.2 at Week 4. The observed increase in SBM frequency with Elo was comparable to that observed in the lubiprostone study (~1.7 vs. ~1.5 times/week).^36^

The effectiveness of Elo, however, was not significant compared to the SBM change observed in the Pbo group, since the estimated difference between the treatment groups was 0.8 with 95% CI of −0.57 to 2.09. The lack of significance of the primary endpoint was likely because of the high SBM frequency in the Pbo group compared to that reported in previous studies. Each patient enrolled in the present study was the first to record daily SBM frequency using a BMD, and this experience might have led to a bias of apparent improvement even in the Pbo group.

Subgroup analyses showed an improvement in SBM frequency in the Pbo group patients, with significance for the two subgroup categories, but none of them was linked to a significant difference between the Elo and Pbo groups. Although a similar situation was seen for more subgroup categories in the Elo group patients, the within‐group significant difference for the subgroup of “prior LEDD < the median (560 mg)" was an exception linked to the between‐group difference for the superiority of Elo to Pbo. This result suggests that the SBM‐normalizing effect of Elo is more apparent in patients with less severe PD.

In the previous phase 3 study of Elo involving patients with chronic constipation, a significant improvement in SBM frequency was observed in the Elo group over the Pbo group at both Weeks 1 and 2.^15^ The discrepancy between the previous and present studies is presumably partly because of the patient definitions applied. For example, the phase 3 study recruited constipated patients who met a symptom‐based criterion of SBM frequency <3 per week and related Rome III definitions.^38^ Such definitions were not prerequisite in the present study, though only a part of the patient criteria.^16^ The age distribution was rather older in the present study, possibly affecting the results. The proportion of Elo group patients who used rescue medicine during the observation period was higher in the present study than in the phase 3 study (~29% vs. ~15%).^15^ This suggests that the patients had relatively severe constipation in the present study.

Examining secondary endpoints (changes in SBM frequency from baseline at Weeks 1, 2, 3, and 4), Elo was found to have significant effectiveness. In addition, effectiveness was observed in the frequency of complete SBMs. The between‐group difference was significant for SBM frequency only at Week 1 on ANCOVA analysis after adjustment by baseline value and sex. Elo was similarly shown to be more efficacious than Pbo for complete SBMs/week at Weeks 1 and 2.

The disappearance of a between‐group difference at Week 4 might be explained by study drug usage. Unlike the usage observed in the previous phase 3 studies, the present study allowed dose increases or decreases of Elo and Pbo according to the patient's judgment. The proportion of patients who reduced the drug dose was 23.7% in the Elo group, but only 5.1% in the Pbo group. The Elo group patients who reported an improvement in SBM frequency at Weeks 1 and/or 2 were likely to have reduced the drug dose during the later treatment period. The dose reduction would underlie the lack of a significant between‐group difference in SBM frequency at Week 4.

Constipation often deteriorates the QOL of afflicted patients. Since a correlation of patients’ QOL with stool form was previously reported,^39^ and stool form was indeed improved by Elo treatment in the present study, multiple QOL indices were further examined. Significant effectiveness of Elo over Pbo was observed for JPAC‐QOL satisfaction and PDQ‐39 stigma subscales. However, unexpectedly, the BSFS‐based subgroup analysis showed significant improvement of the satisfaction score in the patients with not type 1/2, but type 3–5 baseline BSFS. The mean scores were generally higher at baseline in the type 1/2 patients in the Pbo group (see Table S7). The QOL might have tended to improve from their poor baseline status in these patients during the 4‐week treatment period, as contrasted with type 3–5 patients who achieved minor changes in the QOL scores during the period. This presumably led to an insignificant between‐group difference in type 1/2 patients. Despite the considerations above, Elo appears useful for QOL improvement, as the satisfaction score was comparably improved in type 1/2 and type 3–5 patients. Stigma was also improved in the Elo group. The MDS‐UPDRS total score improved significantly in the Pbo group, indicating that a positive change occurred in the PD condition even in this group. The impact of this result on the entire study was unclear.

In consideration of the recent trend of patient‐reported outcomes, QOL improvement is a key issue in the treatment of constipation.^40^, ^41^ Though it was not reported in the lubiprostone study,^36^ the stool form‐QOL correlation may highlight the benefit associated with the use of Elo.

During the treatment period, there were no large differences in the use of rescue medication or L‐dopa and related drugs between the Elo and Pbo groups. More than half of Elo group patients reported previously known AEs related to gastrointestinal disorders, and none of them was of severe intensity. Thus, the AE profile observed in the present study was within the expected range, raising no new safety concerns about Elo. Furthermore, the tolerability of Elo appeared acceptable, because only one patient discontinued the study in the Elo group.

Overall, the present study has strengths: it showed the effectiveness of Elo in increasing the frequency of SBMs as well as complete SBMs, improving stool form, and improving patients’ satisfaction and stigma with treatment. However, the present data lacked robustness, since the drug's effectiveness was not thoroughly demonstrated when compared with Pbo, except for part of the data in a subgroup analysis. The targeted sample size of 40 patients in each group was not met in our study, likely leading to the observed lack of consistency in the effectiveness of Elo. An improving tendency in QOL surveillance observed in Pbo group patients was another limitation, showing limited detection of the benefit of Elo treatment. The weakness might have been resolved if the set definition of SBM frequency was a prerequisite for patient inclusion (see above), more patients were enrolled, or patient demographic characteristics were comparable to those observed in the previous studies. Additionally, co‐morbidities were collectively, not separately, recorded throughout the study period.

In general, Elo has been accepted as a useful drug for the treatment of chronic, even year‐lasting constipation.^14^ However, no reports describing its effectiveness and safety profile in PD patients with chronic constipation have been available to date. Our report is the first one in this sense, and will provide suggestions for further studies of Elo including those to be conducted to examine the long‐term profile of the drug.

In conclusion, Elo improved weekly SBM frequency, though the primary endpoint was not evidenced. QOL parameters (stool consistency and treatment satisfaction) were also improved by the drug. Elo may thus have potential, therapeutic benefits in PD patients with chronic constipation.

## Author Roles

(1) Research project: A. Conception, B. Organization, C. Execution; (2) Statistical Analysis: A. Design, B. Execution, C. Review and Critique; (3) Manuscript Preparation: A. Writing of the first draft, B. Review and Critique.

T.H.: 1A, 1B, 1C, 2B, 2C, 3A.

G.O.: 1A, 1B, 1C, 3B.

Y.S.: 1C, 3B.

K.O.: 1C, 3B.

N.N.: 1C, 3B.

R.N.: 1C, 3B.

T.T.: 1C, 3B.

T.O.: 1C, 3B.

H.E. 1C, 3B.

K.D.: 1C, 3B.

N.K.: 1C, 3B.

S.U.: 1C, 3B.

J.F.: 1C, 3B.

W.S.: 1C, 3B.

K.S.: 1C, 3B.

S.N.: 1C, 3B.

Y.O.: 1C, 3B.

R.W.: 1C, 3B.

S.S.: 1C, 3B.

K.N.: 1C, 3B.

A.O.: 1C, 3B.

D.T.: 1C, 3B.

H.T‐A.: 1C, 3B.

A.F.: 1C, 3B.

A.N.: 1C, 3B.

M.K.: 1C, 3B.

H.K.: 1C, 3B.

Y.Y.: 1C, 3B.

A.S.: 1C, 3B.

N.Y.: 2B, 2C, 3B.

N.H.: 1A, 1B, 1C, 3B.

## Disclosures

**Ethical Compliance Statement:** This study was approved by the Juntendo University Certified Review Board (CRB3180012) and conducted in accordance with the Declaration of Helsinki, the Clinical Trials Act of the Japan Ministry of Health, Labour and Welfare, and related laws and regulations. The participating patients were informed of the study details and provided their written, informed consent. We confirm that we have read the Journal's position on issues involved in ethical publication and affirm that this work is consistent with those guidelines.

**Funding Sources and Conflicts of Interest:** The present study was funded by EA Pharma Co., Ltd. (Tokyo, Japan) and Mochida Pharmaceutical Co., Ltd. (Tokyo, Japan). The authors declare that there are no conflicts of interest relevant to this work.

**Financial Disclosures for the Previous 12 Months:** T.H. reports receiving grants from Kyowa Kirin Co., Ltd., the Setsuro Fujii Memorial, the Osaka Foundation for Promotion of Fundamental Medical Research, JSPS KAKENHI (grant number 21K07424), the Japan Agency for Medical Research and Development (grant numbers 21wm0425015 and 21dk0207055), and Daiichi Sankyo Selects Research Partners for TaNeDS Collaborative Drug Discovery Project; and speakers’ honoraria from Sumitomo Dainippon Pharma Co. Ltd., Takeda Pharmaceutical Co. Ltd., Kyowa Kirin Co. Ltd., Novartis Pharma K.K., Sanofi K.K., Eisai Co. Ltd., and Otsuka Pharmaceutical Co. Ltd. during the conduct of the study. G.O. reports receiving a grant from the Japan Society for the Promotion of Science, a Grant‐in‐Aid for Scientific Research (C) (grant number 21K12711); and speaker honoraria from Medtronic, Boston Scientific, Otsuka Pharmaceutical Co. Ltd., Sumitomo Dainippon Pharma Co. Ltd., Eisai Co. Ltd., Takeda Pharmaceutical Company Ltd., Kyowa Hakko Kirin Co. Ltd., and AbbVie, Inc. Y.S. reports receiving a grant from JSPS KAKENHI (grant number 21K07282) and receiving speakers’ honoraria from Medtronic, Boston Scientific, Otsuka Pharmaceutical Co. Ltd., Takeda Pharmaceutical Co. Ltd., Sumitomo Dainippon Pharma Co. Ltd., Novartis Pharma K.K., MSD, FP Pharmaceutical Corporation, Kyowa Hakko Kirin Co. Ltd., and AbbVie, Inc. K.O. reports receiving a grant from JSPS KAKENHI (grant number 19K17047); and speakers’ honoraria from Sumitomo Dainippon Pharma Co. Ltd., Takeda Pharmaceutical Co. Ltd., Kyowa Kirin Co. Ltd., Eisai Co. Ltd., Otsuka Pharmaceutical Co. Ltd., Ono Pharmaceutical Co. Ltd., Fujimoto Pharmaceutical Co. Ltd., Mochida Pharmaceutical Co. Ltd., Daiichi Sankyo Co. Ltd., and Eli Lilly Japan K.K. during the conduct of the study. N.N. reports no disclosures relevant to the manuscript. R.N. reports receiving speaker honoraria from Eisai Co. Ltd. and AbbVie GK. T.T. reports receiving grants from JSPS KAKENHI (grant numbers 18K07510 and 21K07444); speaker honoraria from Daiichi Sankyo Co. Ltd., Eisai Pharmaceutical Co. Ltd., Sumitomo Dainippon Pharma Co. Ltd., AbbVie, Inc., and Takeda Pharmaceutical Co. Ltd. T.O. reports no disclosures relevant to the manuscript. H.E. reports no disclosures relevant to the manuscript. K.D. reports no disclosures relevant to the manuscript. N.K. reports no disclosures relevant to the manuscript. S.U. reports no disclosures relevant to the manuscript. J.F. reports no disclosures relevant to the manuscript. W.S. reports receiving a grant from JSPS KAKENHI (grant number 20K12670); speakers’ honoraria from Sumitomo Dainippon Pharma Co. Ltd., Takeda Pharmaceutical Co. Ltd., Kyowa Kirin Co. Ltd., Eisai Co. Ltd., Bial, Daiichi Sankyo Co. Ltd., and Abbvie GK during the conduct of the study. K.S. reports no disclosures relevant to the manuscript. S.N. reports receiving a grant from JSPS KAKENHI (grant number 22K15718). Y.O. reports no disclosures relevant to the manuscript. R.W. reports no disclosures relevant to the manuscript. S.S. reports no disclosures relevant to the manuscript. K.N. reports no disclosures relevant to the manuscript. A.O. reports receiving a Research Grant for Biogenic Amines and Neurological Disorders, JSPS KAKENHI (grant number 19K16928); and speakers’ honoraria from Takeda Pharmaceutical Co. Ltd. and Kyowa Kirin Co. Ltd. during the conduct of the study. D.T. reports receiving grants from JSPS KAKENHI (grant numbers 21K20696 and 21H04820) during the conduct of the study. H.T‐A. reports receiving a grant from JSPS KAKENHI (grant number 21K20698). A.F. reports receiving speakers’ honoraria from Eisai Co. Ltd. during the conduct of the study. A.N. reports receiving a grant from JSPS KAKENHI (grant number 21K15751) and speakers’ honoraria from Sumitomo Dainippon Pharma Co. Ltd., Takeda Pharmaceutical Co. Ltd., Kyowa Kirin Co. Ltd., Boston Scientific Corporation, Medtronic, Inc., AbbVie GK, and Ono Pharmaceutical Co. Ltd. during the conduct of the study. M.K. reports no disclosures relevant to the manuscript. H.K. reports receiving speakers’ honoraria from AbbVie GK during the conduct of the study. Y.Y. reports receiving grants from JSPS KAKENHI (grant numbers 19K19951 and 22K17810). A.S. reports no disclosures relevant to the manuscript. N.Y. reports receiving a grant from JSPS KAKENHI (grant number 21K12800). N.H. reports receiving the following grants and fees unrelated to this research during the conduct of the study: grants from the Japan Society for the Promotion of Science (JSPS), the Japan Agency for Medical Research and Development (AMED), the Japan Science and Technology Agency (JST), a Health Labour Sciences Research Grant, IPMDS, and MJFF; personal fees and speakers’ honoraria from Sumitomo Dainippon Pharma Co. Ltd., Takeda Pharmaceutical Co. Ltd., Kyowa Kirin Co. Ltd., AbbVie GK, Otsuka Pharmaceutical Co. Ltd., Novartis Pharma Co. Ltd., Ono Pharmaceutical Co. Ltd., Eisai Co. Ltd., Teijin Pharma, Daiichi Sankyo Co., Ltd., and FP Pharmaceutical Corporation; personal fees for consultancies and advisory boards from Sumitomo Dainippon Pharma Co. Ltd., Takeda Pharmaceutical Co. Ltd., Kyowa Kirin Co. Ltd., Ono Pharmaceutical Co. Ltd., Teijin Pharma, and PARKINSON Laboratories Co. Ltd.; and he owns shares in the PARKINSON Laboratories Co. Ltd. (Equity stock (8%)).

## Supporting information


**Figure S1.** Changes in spontaneous bowel movements during the 4‐week treatment period. Changes in per‐week frequency of SBM (Panel A) and complete SBM (Panel B) from baseline (Week 0) are shown for each treatment week. p values in the figure represent within‐group comparisons vs. Week 0, calculated by paired *t* test. When frequency changes were compared between the Elo and Pbo groups at each week using an ANCOVA model, the statistical significance was *P* = 0.0011, 0.6457, 0.3455, and 0.2340 (Panel A), and *P* = 0.0282, 0.0154, 0.0535, and 0.7595 (Panel B), respectively. The significant p values are annotated with asterisks in the figure. SBM, spontaneous bowel movements; Elo, elobixibat; Pbo, placebo; ANCOVA, analysis of covariance.

**Table S1.** Subgroup analysis of Week 4 vs. baseline changes in per‐week spontaneous bowel movements.
**Table S2.** Between‐treatment difference of Week 4 vs. baseline changes in spontaneous bowel movements: subgroup analysis.
**Table S3.** Changes in per‐week frequencies of spontaneous bowel movements and complete spontaneous bowel movements at each treatment week vs. Week 0.
**Table S4.** Evaluation of stool form changes after BSFS scaling as continuous values.
**Table S5.** Summary of QOL results.
**Table S6.** Week 4 vs. baseline comparison of JPAC‐QOL surveillance by type category of baseline stool form.
**Table S7.** JPAC‐QOL by baseline stool form.
**Table S8.** Adverse events reported during the study period.

## Data Availability

Anonymized data not published within this article will be made available on request from any qualified investigators.
