# Supplementary figures and images for: Efficacy and Safety of Elobixibat in Parkinson's Disease with Chronic Constipation: CONST‐PD Study
Source: Mov Disord Clin Pract. 2024 Jan 24;11(4):352–62. doi: 10.1002/mdc3.13972 (PMC10982595; doi:10.1002/mdc3.13972)

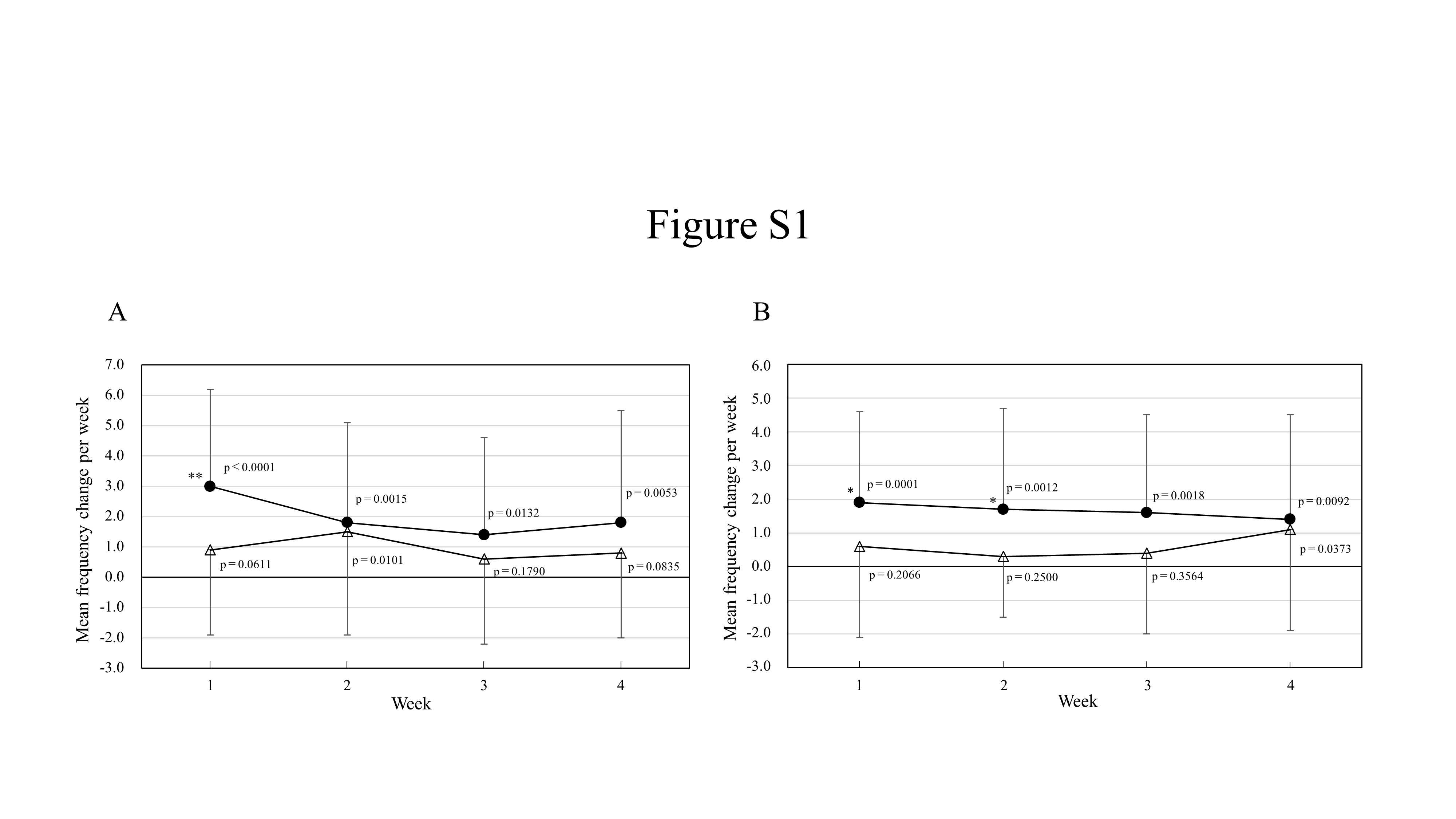

Supplement: Supplementary file 1 — Figure S1. Changes in spontaneous bowel movements during the 4‐week treatment period. Changes in per‐week frequency of SBM (Panel A) and complete SBM (Panel B) from baseline (Week 0) are shown for each treatment week. p values in the figure represent within‐group comparisons vs. Week 0, calculated by paired t test. When frequency changes were compared between the Elo and Pbo groups at each week using an ANCOVA model, the statistical significance was P = 0.0011, 0.6457, 0.3455, and 0.2340 (Panel A), and P = 0.0282, 0.0154, 0.0535, and 0.7595 (Panel B), respectively. The significant p values are annotated with asterisks in the figure. SBM, spontaneous bowel movements; Elo, elobixibat; Pbo, placebo; ANCOVA, analysis of covariance. [file MDC3-11-352-s001.tif]
