# Supplementary material for: Efficacy and Safety of Elobixibat in Parkinson's Disease with Chronic Constipation: CONST‐PD Study
Source: Mov Disord Clin Pract. 2024 Jan 24;11(4):352–62. doi: 10.1002/mdc3.13972 (PMC10982595; doi:10.1002/mdc3.13972)
Supplement: Supplementary file 2 — Table S1. Subgroup analysis of Week 4 vs. baseline changes in per‐week spontaneous bowel movements. Table S2. Between‐treatment difference of Week 4 vs. baseline changes in spontaneous bowel movements: subgroup analysis. Table S3. Changes in per‐week frequencies of spontaneous bowel movements and complete spontaneous bowel movements at each treatment week vs. Week 0. Table S4. Evaluation of stool form changes after BSFS scaling as continuous values. Table S5. Summary of QOL results. Table S6. Week 4 vs. baseline comparison of JPAC‐QOL surveillance by type category of baseline stool form. Table S7. JPAC‐QOL by baseline stool form. Table S8. Adverse events reported during the study period. [file MDC3-11-352-s002.docx]

| **Table S1. Subgroup analysis of Week 4 vs. baseline changes in per-week spontaneous bowel movements** | | | | | |
| --- | --- | --- | --- | --- | --- |
| **Subgroup** |  | **Treatment group** | **n** | **Mean ± SD** | ***p* value** |
| Complications | Yes | Elo | 31 | 1.2 ± 3.6 | 0.0665 |
|  |  | Pbo | 28 | 0.5 ± 3.1 | 0.4108 |
|  | No | Elo | 7 | 3.7 ± 3.7 | 0.0388 |
|  |  | Pbo | 11 | 1.5 ± 1.9 | 0.0233 |
| Age (y) | ≥65 | Elo | 28 | 1.6 ± 3.6 | 0.0224 |
|  |  | Pbo | 19 | 1.4 ± 3.4 | 0.0937 |
|  | <65 | Elo | 10 | 1.9 ± 4.4 | 0.2036 |
|  |  | Pbo | 20 | 0.2 ± 2.1 | 0.6300 |
| Hoehn and Yahr stage | I | Elo | 3 | 5.0 ± 4.6 | 0.1994 |
|  |  | Pbo | 1 | -2.0 | - |
|  | II | Elo | 25 | 1.4 ± 3.5 | 0.0515 |
|  |  | Pbo | 35 | 0.8 ± 2.9 | 0.1059 |
|  | III | Elo | 7 | 1.8 ± 4.6 | 0.3439 |
|  |  | Pbo | 3 | 1.3 ± 1.2 | 0.1835 |
|  | IV | Elo | 3 | 0.3 ± 2.1 | 0.8075 |
|  |  | Pbo | - | - | - |
| Duration of PD (y) | < median | Elo | 20 | 1.6 ± 4.2 | 0.0976 |
|  |  | Pbo | 16 | 2.1 ± 3.0 | 0.0143 |
|  | ≥ median | Elo | 18 | 1.8 ± 3.3 | 0.0345 |
|  |  | Pbo | 23 | -0.1 ± 2.3 | 0.8254 |
| LEDD at baseline (mg) | < median | Elo | 19 | 2.6 ± 4.3 | 0.0175 |
|  |  | Pbo | 18 | 0.6 ± 2.2 | 0.2418 |
|  | ≥ median | Elo | 19 | 0.8 ± 2.9 | 0.2363 |
|  |  | Pbo | 21 | 0.9 ± 3.3 | 0.2173 |
| Duration of chronic constipation (y) | ≥20 | Elo | 7 | 1.4 ± 3.6 | 0.3559 |
|  |  | Pbo | 6 | 1.8 ± 1.8 | 0.0581 |
|  | <20 | Elo | 31 | 1.8 ± 3.8 | 0.0146 |
|  |  | Pbo | 32 | 0.7 ± 2.8 | 0.1544 |
| Week 4 vs. baseline changes in per-week SBM frequency were summarized by subgroup. Median values were 6 years for PD duration and 560 mg/day for LEDD. The same values apply in the following Table S2.  Elo, elobixibat; Pbo, placebo; PD, Parkinson’s disease; SBM, spontaneous bowel movement; SD, standard deviation; LEDD, L-dopa equivalent daily dose. | | | | | |

| **Table S2. Between-treatment difference of Week 4 vs. baseline changes in spontaneous bowel movements: subgroup analysis** | | | |
| --- | --- | --- | --- |
| **Subgroup** |  | **Point estimate (95% CI)** | ***p* value** |
| Complications | Yes | 0.5 (-1.04, 2.06) | 0.5189 |
|  | No | 1.9 (-0.51, 4.38) | 0.1200 |
| Age (y) | ≥65 | 0.0 (-1.87, 1.86) | 0.9937 |
|  | <65 | 2.0 (0.12, 3.92) | 0.0370 |
| Hoehn and Yahr stage | I | 5.0 (1.08, 8.92) | 0.0124 |
|  | II | 0.5 (-0.99, 1.95) | 0.5213 |
|  | III | 1.9 (-1.03, 4.85) | 0.2039 |
|  | IV | 0.0 (0.00, 0.00) | - |
| Duration of PD (y) | < median | -0.2 (-2.29, 1.79) | 0.8133 |
|  | ≥ median | 1.6 (-0.05, 3.15) | 0.0571 |
| LEDD at baseline (mg) | < median | 1.8 (0.08, 3.62) | 0.0401 |
|  | ≥ median | -0.2 (-2.01, 1.67) | 0.8577 |
| Duration of chronic constipation (y) | ≥20 | -0.7 (-3.34, 1.97) | 0.6140 |
|  | <20 | 1.0 (-0.53, 2.45) | 0.2080 |
| The difference in per-week SBM frequency changes was compared by subgroup between the Elo and Pbo groups after adjustment with baseline values and sex.  CI, confidence interval; Elo, elobixibat; Pbo, placebo; SBM, spontaneous bowel movement; LEDD, L-dopa equivalent daily dose. | | | |

**Table S3 Changes in per-week frequencies of spontaneous bowel movements and complete spontaneous bowel movements at each treatment week vs. Week 0**

| **Treatment week** | | **1** | **2** | **3** | **4** |
| --- | --- | --- | --- | --- | --- |
| SBM | Elo (mean ± SD) | 3.0 ± 3.2 | 1.8 ± 3.3 | 1.4 ± 3.2 | 1.8 ± 3.7 |
|  | Pbo (mean ± SD) | 0.9 ± 2.8 | 1.5 ± 3.4 | 0.6 ± 2.8 | 0.8 ± 2.8 |
| CSBM | Elo (mean ± SD) | 1.9 ± 2.7 | 1.7 ± 3.0 | 1.6 ± 2.9 | 1.4 ± 3.1 |
|  | Pbo (mean ± SD) | 0.6 ± 2.7 | 0.3 ± 1.8 | 0.4 ± 2.4 | 1.1 ± 3.0 |

Values are presented as frequency per week. *p* values for within-group comparisons at each week vs. Week 0 were noted in Figure. S1. See the Figure. S1 legend for statistical significance for Elo vs. Pbo group comparisons at each week.

SBM, spontaneous bowel movement; CSBM, complete spontaneous bowel movement; SD, standard deviation; Elo, elobixibat; Pbo, placebo.

**Table S4. Evaluation of stool form changes after BSFS scaling as continuous values**

|  |  | **Elobixibat** | **Placebo** | ***p* value** |
| --- | --- | --- | --- | --- |
| Baseline | N | 37 | 39 |  |
|  | Mean (SD) | 2.5 (1.3) | 2.7 (1.4) | 0.5692 |
| Week 1 | N | 38 | 39 |  |
|  | Mean (SD) | 3.9 (1.6) | 2.8 (1.6) | 0.0057 |
| Week 2 | N | 38 | 39 |  |
|  | Mean (SD) | 4.0 (1.4) | 3.0 (1.4) | 0.0119 |
| Week 3 | N | 37 | 38 |  |
|  | Mean (SD) | 3.5 (1.2) | 3.2 (1.4) | 0.4461 |
| Week 4 | N | 36 | 38 |  |
|  | Mean (SD) | 3.8 (1.6) | 3.1 (1.5) | 0.1035 |
| Between-group differences were examined by the Wilcoxon rank-sum test.  BSFS, Bristol Stool Form Scale; SD, standard deviation. | | | | |

| **Table S5. Summary of QOL results** | | | | | |
| --- | --- | --- | --- | --- | --- |
| **QOL index** |  |  | **Elobixibat** | **Placebo** | ***p* value** |
| MDS-UPDRS | Part I subtotal | Baseline | 10.1 ± 4.7 | 10.0 ± 4.6 | 0.9206 |
|  |  | Week 4 | 9.1 ± 4.8 | 9.6 ± 4.4 | 0.6327 |
|  | Part II subtotal | Baseline | 11.1 ± 7.7 | 11.9 ± 7.5 | 0.6474 |
|  |  | Week 4 | 11.8 ± 8.0 | 11.6 ± 7.5 | 0.9168 |
|  | Part III subtotal | Baseline | 19.1 ± 12.0 | 17.9 ± 8.8 | 0.6300 |
|  |  | Week 4 | 17.4 ± 11.0 | 14.4 ± 6.9 | 0.1672 |
|  | Part IV subtotal | Baseline | 2.8 ± 3.3 | 2.4 ± 3.7 | 0.6117 |
|  |  | Week 4 | 2.8 ± 3.5 | 2.5 ± 3.6 | 0.7919 |
|  | Total | Baseline | 43.2 ± 22.8 | 42.3 ± 18.4 | 0.8527 |
|  |  | Week 4 | 41.1 ± 20.9 | 38.3 ± 15.0 | 0.5008 |
| PDQ-39 | Mobility | Baseline | 10.4 ± 9.8 | 10.1 ± 8.6 | 0.8993 |
|  |  | Week 4 | 9.1 ± 9.5 | 8.9 ± 9.4 | 0.9317 |
|  | Activity of daily living | Baseline | 4.1 ± 4.5 | 5.5 ± 5.0 | 0.1742 |
|  |  | Week 4 | 3.8 ± 4.6 | 5.1 ± 5.3 | 0.2364 |
|  | Emotional well-being | Baseline | 4.3 ± 4.1 | 4.9 ± 4.2 | 0.5041 |
|  |  | Week 4 | 3.6 ± 3.5 | 4.8 ± 4.3 | 0.1985 |
|  | Stigma | Baseline | 2.2 ± 2.5 | 2.4 ± 2.9 | 0.8135 |
|  |  | Week 4 | 1.6 ± 2.4 | 2.8 ± 3.3 | 0.0663 |
|  | Communication | Baseline | 0.9 ± 1.6 | 1.0 ± 2.0 | 0.8989 |
|  |  | Week 4 | 0.7 ± 1.4 | 0.8 ± 1.9 | 0.8901 |
|  | Bodily discomfort | Baseline | 3.4 ± 2.4 | 3.4 ± 2.9 | 0.9785 |
|  |  | Week 4 | 3.2 ± 2.6 | 3.4 ± 2.8 | 0.7634 |
|  | Social support | Baseline | 1.6 ± 1.6 | 1.9 ± 1.9 | 0.4283 |
|  |  | Week 4 | 1.5 ± 2.2 | 1.8 ± 1.9 | 0.5692 |
|  | Cognition | Baseline | 2.9 ± 3.0 | 2.4 ± 2.4 | 0.4115 |
|  |  | Week 4 | 2.8 ± 3.2 | 2.8 ± 2.8 | 1.0000 |
|  | Total | Baseline | 29.7 ± 23.1 | 31.6 ± 22.2 | 0.7173 |
|  |  | Week 4 | 26.2 ± 23.3 | 30.4 ± 24.9 | 0.4595 |
| EQ-5D | Index value | Baseline | 0.7 ± 0.2 | 0.7 ± 0.2 | 0.6481 |
|  |  | Week 4 | 0.7 ± 0.2 | 0.7 ± 0.2 | 0.8628 |
|  | VAS | Baseline | 70.7 ± 15.6 | 68.0 ± 16.1 | 0.4473 |
|  |  | Week 4 | 67.2 ± 18.7 | 68.3 ± 15.7 | 0.7835 |

Values for each treatment group are expressed as mean ± SD. The Elo and Pbo groups included 38 and 39 patients, respectively, at baseline, and 37 each at Week 4.

EQ-5D, Euro-Qol 5 dimensions; MDS-UPDRS, Movement Disorder Society-unified Parkinson’s Disease Rating Scale; PDQ-39, Parkinson’s Disease Questionnaire-39; QOL, quality of life; SD, standard deviation; VAS, visual analogue scale.

| **Table S6. Week 4 vs. baseline comparison of JPAC-QOL surveillance by type category of baseline stool form** | | | | |
| --- | --- | --- | --- | --- |
| **Baseline BSFS type** | **JPAC-QOL subscale** | **Elo** | **Pbo** | ***p* value** |
| 1/2 | Physical discomfort | -0.4 ± 0.7 | 0.1 ± 0.7 | 0.0550 |
|  | Psychosocial discomfort | -0.1 ± 0.4 | -0.2 ± 0.4 | 0.7118 |
|  | Worries/concerns | 0.0 ± 0.4 | -0.1 ± 0.5 | 0.3835 |
|  | Satisfaction | -0.6 ± 1.1 | -0.2 ± 0.8 | 0.1951 |
|  | Total | -0.2 ± 0.4 | -0.1 ± 0.4 | 0.5668 |
| 3-5 | Physical discomfort | -0.2 ± 1.0 | 0.1 ± 0.7 | 0.4505 |
|  | Psychosocial discomfort | -0.1 ± 0.6 | 0.0 ± 0.4 | 0.7328 |
|  | Worries/concerns | -0.1 ± 0.8 | 0.0 ± 0.5 | 0.7848 |
|  | Satisfaction | -0.7 ± 1.2 | 0.2 ± 0.8 | 0.0158 |
|  | Total | -0.2 ± 0.7 | 0.0 ± 0.5 | 0.2456 |
| Values in the treatment group columns are expressed as mean ± SD. *p* values were calculated by the 2-sample *t-*test. The numbers of patients evaluated were 18 (Elo group) and 17 (Pbo group) for BSFS type 1/2, and 18 (Elo group) and 20 (Pbo group) for BSFS type 3-5.  BSFS, Bristol Stool Form Scale; Elo, elobixibat; JPAC-QOL, Japanese version of Patient Assessment of Constipation Quality of Life; Pbo, placebo; SD, standard deviation. | | | | |

| **Table S7. JPAC-QOL by baseline stool form** | | | | | |
| --- | --- | --- | --- | --- | --- |
| **Baseline BSFS** | **Subscale** |  | **Elobixibat** | **Placebo** | ***p* value** |
| All types | Physical discomfort | Baseline | 1.3 ± 0.7 | 1.3 ± 0.8 | 0.9094 |
|  |  | Week 4 | 1.0 ± 0.6 | 1.3 ± 0.8 | 0.0546 |
|  | Psychosocial discomfort | Baseline | 0.6 ± 0.6 | 0.6 ± 0.5 | 0.9537 |
|  |  | Week 4 | 0.5 ± 0.5 | 0.5 ± 0.4 | 0.8968 |
|  | Worries/concerns | Baseline | 1.0 ± 0.7 | 1.1 ± 0.7 | 0.7884 |
|  |  | Week 4 | 0.9 ± 0.6 | 0.9 ± 0.6 | 0.9709 |
|  | Satisfaction | Baseline | 2.8 ± 0.6 | 2.8 ± 0.7 | 0.9246 |
|  |  | Week 4 | 2.2 ± 1.0 | 2.9 ± 0.9 | 0.0022 |
|  | Total | Baseline | 1.2 ± 0.5 | 1.3 ± 0.6 | 0.8456 |
|  |  | Week 4 | 1.0 ± 0.5 | 1.2 ± 0.5 | 0.1359 |
| Type 1/2 | Physical discomfort | Baseline | 1.1 ± 0.7 | 1.4 ± 0.8 | 0.3422 |
|  |  | Week 4 | 0.8 ± 0.5 | 1.5 ± 0.8 | 0.0039 |
|  | Psychosocial discomfort | Baseline | 0.5 ± 0.5 | 0.8 ± 0.8 | 0.1077 |
|  |  | Week 4 | 0.4 ± 0.4 | 0.6 ± 0.4 | 0.0416 |
|  | Worries/concerns | Baseline | 0.8 ± 0.5 | 1.3 ± 0.8 | 0.0276 |
|  |  | Week 4 | 0.7 ± 0.3 | 1.1 ± 0.6 | 0.0455 |
|  | Satisfaction | Baseline | 2.7 ± 0.5 | 3.2 ± 0.5 | 0.0068 |
|  |  | Week 4 | 2.1 ± 0.9 | 3.0 ± 0.8 | 0.0029 |
|  | Total | Baseline | 1.1 ± 0.4 | 1.5 ± 0.6 | 0.0223 |
|  |  | Week 4 | 0.9 ± 0.4 | 1.4 ± 0.5 | 0.0029 |
| Type 3-5 | Physical discomfort | Baseline | 1.3 ± 0.7 | 1.2 ± 0.8 | 0.5309 |
|  |  | Week 4 | 1.2 ± 0.6 | 1.2 ± 0.8 | 0.8937 |
|  | Psychosocial discomfort | Baseline | 0.6 ± 0.6 | 0.4 ± 0.4 | 0.1958 |
|  |  | Week 4 | 0.6 ± 0.6 | 0.4 ± 0.4 | 0.2032 |
|  | Worries/concerns | Baseline | 1.2 ± 0.8 | 0.9 ± 0.7 | 0.1996 |
|  |  | Week 4 | 1.1 ± 0.7 | 0.8 ± 0.5 | 0.1357 |
|  | Satisfaction | Baseline | 2.9 ± 0.7 | 2.6 ± 0.8 | 0.1466 |
|  |  | Week 4 | 2.3 ± 1.0 | 2.7 ± 0.9 | 0.1444 |
|  | Total | Baseline | 1.4 ± 0.6 | 1.1 ± 0.5 | 0.1416 |
|  |  | Week 4 | 1.2 ± 0.5 | 1.1 ± 0.5 | 0.5548 |
| Values for each treatment group are expressed as mean ± SD. The *p* values were determined by the paired *t-*test. The numbers of patients evaluated were 38 in the Elo group and 39 in the Pbo group at baseline and 37 each at Week 4 for overall JPAC-QOL analysis. For subgroup analysis, the numbers were 18 and 17 at both baseline and Week 4 in the BSFS type 1/2 patients, and in the BSFS type 3-5 patients, the numbers were 19 and 22 at baseline and 18 and 20 at Week 4, respectively.  BSFS, Bristol Stool Form Scale; JPAC-QOL, Japanese version of Patient Assessment of Constipation Quality of Life; SD, standard deviation. | | | | | |

| **Table S8. Adverse events reported during the study period** | | | | | | | | |
| --- | --- | --- | --- | --- | --- | --- | --- | --- |
|  | **Elobixibat** | | | | **Placebo** | | | |
| **SOC/PT** | **Total** | **Intensity** | | | **Total** | **Intensity** | | |
|  |  | **Mild** | **Moderate** | **Severe** |  | **Mild** | **Moderate** | **Severe** |
| Patients at risk (N) | 38 |  |  |  | 39 |  |  |  |
| Patients with AEs, n (%) | 21 (55.3) | 20 (52.6) | 1 (2.6) | 0 (0.0) | 4 (10.3) | 4 (10.3) | 0 (0.0) | 0 (0.0) |
| Gastrointestinal disorders | 21 (55.3) | 20 (52.6) | 1 (2.6) | 0 (0.0) | 4 (10.3) | 4 (10.3) | 0 (0.0) | 0 (0.0) |
| Abdominal discomfort | 0 (0.0) | 0 (0.0) | 0 (0.0) | 0 (0.0) | 1 (2.6) | 1 (2.6) | 0 (0.0) | 0 (0.0) |
| Abdominal pain | 1 (2.6) | 1 (2.6) | 0 (0.0) | 0 (0.0) | 1 (2.6) | 1 (2.6) | 0 (0.0) | 0 (0.0) |
| Constipation | 0 (0.0) | 0 (0.0) | 0 (0.0) | 0 (0.0) | 1 (2.6) | 1 (2.6) | 0 (0.0) | 0 (0.0) |
| Diarrhea | 11 (28.9) | 11 (28.9) | 0 (0.0) | 0 (0.0) | 1 (2.6) | 1 (2.6) | 0 (0.0) | 0 (0.0) |
| Malabsorption | 1 (2.6) | 0 (0.0) | 1 (2.6) | 0 (0.0) | 0 (0.0) | 0 (0.0) | 0 (0.0) | 0 (0.0) |
| Feces soft | 12 (31.6) | 12 (31.6) | 0 (0.0) | 0 (0.0) | 2 (5.1) | 2 (5.1) | 0 (0.0) | 0 (0.0) |
| AEs were collected from baseline to Week 4. Preferred terms were according to MedDRA ver. 25.0 for each reported AE. % was calculated as n/N × 100.  AE, adverse event; Elo, elobixibat; Pbo, placebo; PT, Preferred Term; SOC, System Organ Class. | | | | | | | | |
